# Seroconversions for *Coxiella* and Rickettsial Pathogens among US Marines Deployed to Afghanistan, 2001–2010

**DOI:** 10.3201/eid2208.160221

**Published:** 2016-08

**Authors:** Christina M. Farris, Nhien Pho, Todd E. Myers, Allen L. Richards

**Affiliations:** US Naval Medical Research Unit 2, Phnom Penh, Cambodia (C.M. Farris);; Naval Medical Research Center, Silver Spring, Maryland, USA (C.M. Farris, T.E. Myers, A.L. Richards);; Uniformed Services University of Health Science, Bethesda, Maryland, USA (N. Pho)

**Keywords:** rickettsia, Q fever, *Coxiella*, Afghanistan, deployment, military, bacteria, gram-negative bacteria, rickettsial infections, zoonoses, rickettsiosis, seroconversion

## Abstract

We assessed serum samples from 1,000 US Marines deployed to Afghanistan during 2001–2010 to find evidence of 4 rickettsial pathogens. Analysis of predeployment and postdeployment samples showed that 3.4% and 0.5% of the Marines seroconverted for the causative agents of Q fever and spotted fever group rickettsiosis, respectively.

Rickettsial and rickettsial-like diseases have played a considerable role in military activities throughout much of recorded history (*1*). These diseases, which have worldwide distribution and cause a high number of deaths and illnesses, include the select agents (http://www.selectagents.gov/SelectAgentsandToxinsList.html) *Rickettsia prowazekii* and *Coxiella burnetii*, the causative agents of epidemic typhus and Q fever, respectively. Furthermore, outbreaks caused by rickettsial disease pathogens, such as scrub typhus (*Orientia tsutsugamushi*) in Laos, Vietnam, and Cambodia and African tick-bite fever (*Rickettsia africae*) in Botswana, have affected military forces in recent history (*2*,*3*). *O. tsutsugamushi* was the leading cause of fever of unknown origin in US soldiers during the Vietnam conflict and caused >16,000 cases of scrub typhus among Allied forces during World War II (*1*).

The epidemiology of rickettsial pathogens is not well understood in Central Asia, where >100,000 US and allied troops have been engaged in military operations since 2001. Retrospective studies from military personnel deployed during Operation Desert Shield/Desert Storm in 1991 revealed exposures of 9.8% and 5.7% for spotted fever group rickettsiae (SFGR) and typhus group rickettsiae (TGR), respectively; however, no seroconversions were observed (*4*). Since 2001 in Iraq, >150 cases of Q fever have been confirmed in US troops and civilians (*5*–*8*). An outbreak of Q fever and brucellosis in local residents in the Bamyan province of Afghanistan during 2011 (*9*) and the historic presence of SFGR and vectors known to carry SFGR (*10*) highlight the inherent risk of contracting rickettsial-like diseases in Afghanistan. We estimated the risk for rickettsial infections in military personnel deployed to Afghanistan by measuring the rate of seroconversion for SFGR, TGR, scrub typhus group *Orientiae* (STGO), and *C. burnetii* among US Marines stationed in Afghanistan during 2001–2010.

## The Study 

Serum samples from US Marines 18–45 years of age who served >180 days in Afghanistan during 2001–2010 were obtained from the US Department of Defense Serum Repository (DoDSR). Documentation of prior exposure to Q fever or rickettsioses and sample volume <0.5 mL were exclusion criteria. We selected the most recent 1,000 postdeployment specimens that fit the inclusion criteria for our study.

Military service members have blood drawn every 2 years for HIV testing and during postdeployment screenings, and a portion of each sample is stored at the DoDSR. All predeployment samples were collected <1 year before the start of Afghanistan service, and postdeployment samples were collected within ≈1 year after the end of Afghanistan service. Paired predeployment and postdeployment samples were irreversibly stripped of personal identifiers by DoDSR and labeled with an internal DoDSR code. Only DoDSR has access to the key linking the code to personal identifiers. The Naval Medical Research Center Institutional Review Board approved the study.

We screened specimens for antibodies against TGR, SFGR, and STGO by ELISA, as described (*11*) and determined titers for positive specimens. Seroconversion (i.e., titer <100 in the predeployment sample and titer >400 in the paired postdeployment sample) or a 4-fold rise in titer between the predeployment and paired postdeployment sample was used to determine acute infection.

We tested specimens for antibodies against *C. burnetii* by using the Q fever Immunodot assay (GenBio, San Diego, CA, USA), according to the manufacturer’s instructions. Specimens were considered positive for acute infection if antibodies against phase I and phase II antigen were present. Seroconversion was defined by the presence of antibodies against phase I antigen in the postdeployment sample but not in the predeployment sample.

Of the 1,000 postdeployment serum samples screened for SFGR, TGR, and STGO, only 879 were screened for Q fever because the volume of 121 samples was depleted from earlier testing. The screening assays showed that 87, 18, and 1 samples were positive for antibodies against *C. burnetii*, SFGR, and STGO, respectively, before deployment. No antibodies against TGR were detectable in any samples. Seroconversions to *C. burnetii* and SFGR occurred in 3.4% and 0.5% of the paired serum samples, respectively (Table). *C. burnetii* infection was most prevalent among the agents tested both before deployment (n = 87) and during deployment (n = 30). Of 879 specimens, 117 (13.3%) were positive.

**Table:** Prevalence of antibodies against 4 rickettsial pathogens in samples from US Marines deployed to Afghanistan during 2001–2010

**Pathogen**	Total no. samples tested	No. (%)		
		Predeployment samples with detectable antibody	Postdeployment samples showing seroconversion	Total samples with detectable antibody
***Coxiella burnetii***	879	87 (9.9)	30 (3.4)	117 (13.3)
**Spotted fever group *Rickettsia***	1,000	18 (1.8)	5 (0.5)	23 (2.3)
**Typhus group *Rickettsia***	1,000	0	0	0
***Orientia tsutsugamushi***	3,654	1 (0.1)	0	1 (0.1)

Most of the 30 *C. burnetii* seroconversions occurred in Marines who began deployment in 2008 (n = 12) and 2009 (n = 8) (Figure, panel A). However, most (634/879 [72.1%]) of the sample population were deployed during this period (Figure, panel B), so higher rates for these years likely do not indicate higher risk. With the exception of 1 fixed-wing pilot, all *C. burnetii* and SFGR seroconversions occurred in general infantrymen, who represented 93.1% of *C. burnetii*–positive samples and 92.6% of total samples.

**Figure:**
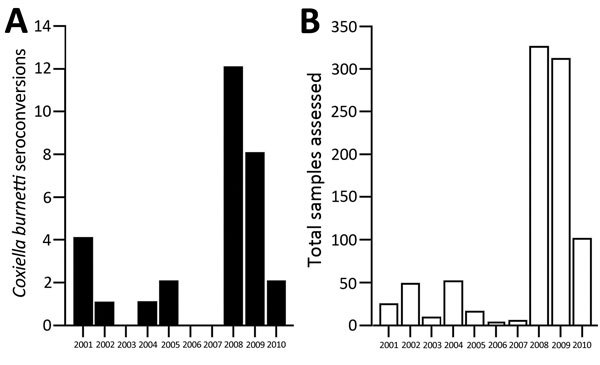
Serum samples assessed for evidence of seroconversion for *Coxiella burnetii* among US Marines deployed to Afghanistan, by year deployment began, 2001–2010. A) Number of *Coxiella burnetii* seroconversions (n = 30). B) Total number of samples assessed for antibodies against *Coxiella burnetii *(n = 879).

Range of deployment time was 180–450 days, and average length of deployment was 271 (SD 104.49) days. Average length of deployment for those who seroconverted for *C. burnetii* or SFGR was 286.2 (SD 112.01) and 342.4 (SD 131.65) days, respectively. Analysis by *t*-test showed no correlation between length of deployment and seroconversion for either *C. burnetii* (p = 0.98) or SFGR (p = 1.0).

Predeployment serum samples were collected 3–364 (mean 183, SD 111.07) days before deployment, and postdeployment samples were collected <443 (mean 61.3, SD 111.02) days after return. Samples showing seroconversions for *C. burnetii* and SFGR were collected an average of 179.3 (SD 93.94) and 151.6 (SD 126.76) days, respectively, predeployment and 76.9 (SD 87.64) and 129.8 (SD 127.85) days, respectively, postdeployment.

## Conclusions

Acute febrile illness can be difficult to diagnose because many infections have similar symptoms and signs and are difficult to differentiate without appropriate diagnostic tools. The rate of *C. burnetii* seroconversions (3.4%) in Marines in our study is similar to that reported in United Kingdom military personnel deployed to Afghanistan during 2008–2011 (*7*); the study from the United Kingdom also confirms the presence of rickettsiae in the region. The rate of *C. burnetii* seropositivity in the United States is ≈3% but ranges dramatically; nearby Nova Scotia, Canada, has rates >14% (*8*,*12*). With proper treatment of infections, the case-fatality rate for Q fever and rickettsial infections is <2%. However, rates can be >30%; among hospitalized Mediterranean spotted fever patients in Portugal in 1997, the case-fatality rate was 32.5% (*13*).

In our study, gaps between dates of blood draws and start and end dates of deployment provided an opportunity for exposure to agents of Q fever and SFGR outside the deployment period. The nature of retrospective, blinded studies prevents follow-up with participants to ascertain additional information, including whether symptoms developed or treatment was sought. This limitation is especially true for studies of military populations, for whom routine blood draws are typically used, rather than samples being specifically collected for research studies. However, results from these studies provide valuable information, and in our study, this limitation does not discount the documented prevalence of Q fever in Iraq and Afghanistan (*4**–**10**,**14*) or the inherent risk to deployed US military personnel. Our results highlight the risk of contracting Q fever and the need for Q fever diagnostics in military engagements in Central Asia.
